# Supplementary material for: IL-17A Is the Critical Cytokine for Liver and Spleen Amyloidosis in Inflammatory Skin Disease
Source: Int J Mol Sci. 2022 May 20;23(10):5726. doi: 10.3390/ijms23105726 (PMC9147816; doi:10.3390/ijms23105726)
Supplement: Supplementary file 1 [file ijms-23-05726-s001.zip › ijms-1679314-supplementary.pptx]

## Slide 1
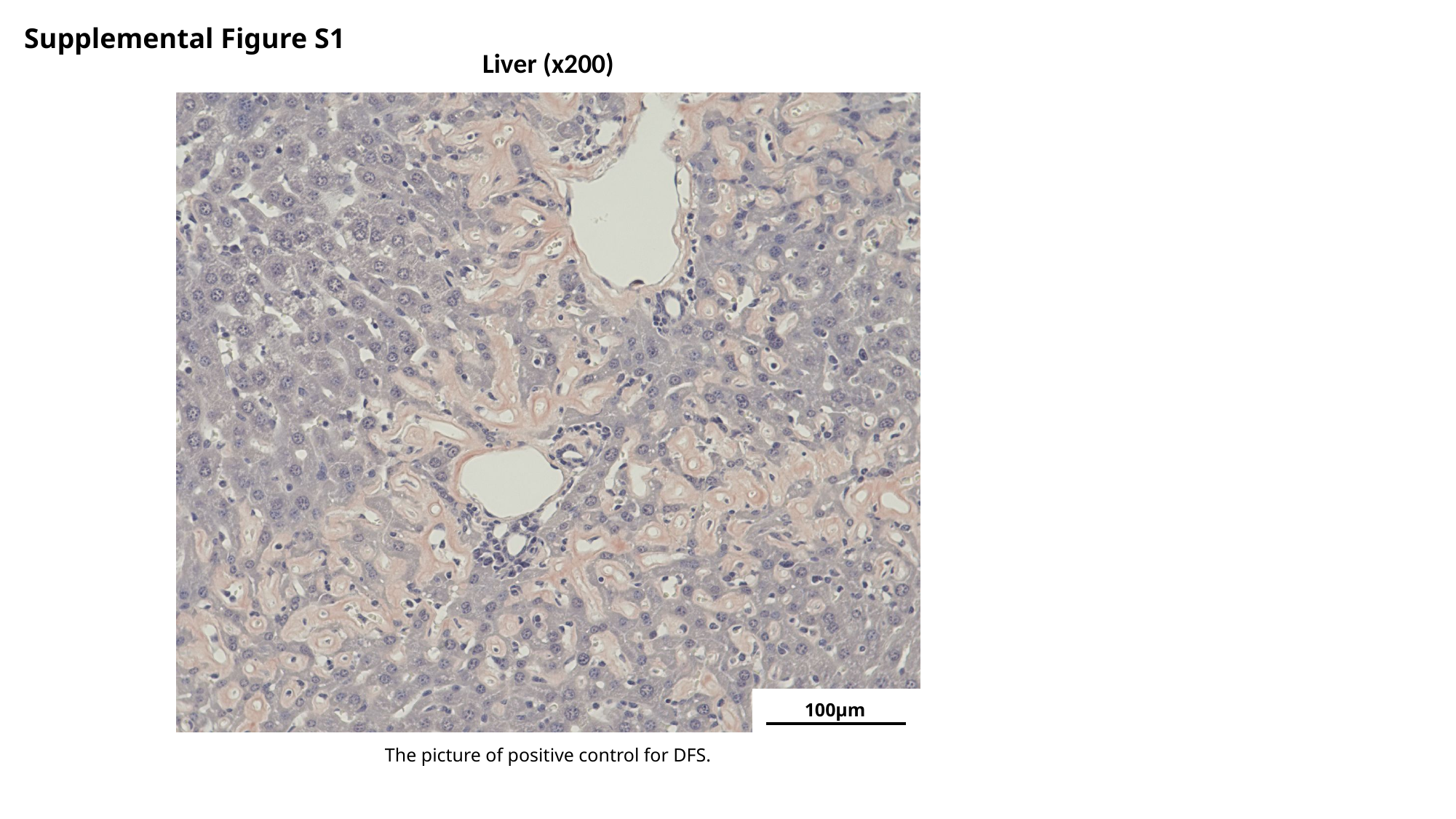

Supplemental Figure S1
Liver (x200)
100μm
The picture of positive control for DFS.

## Slide 2
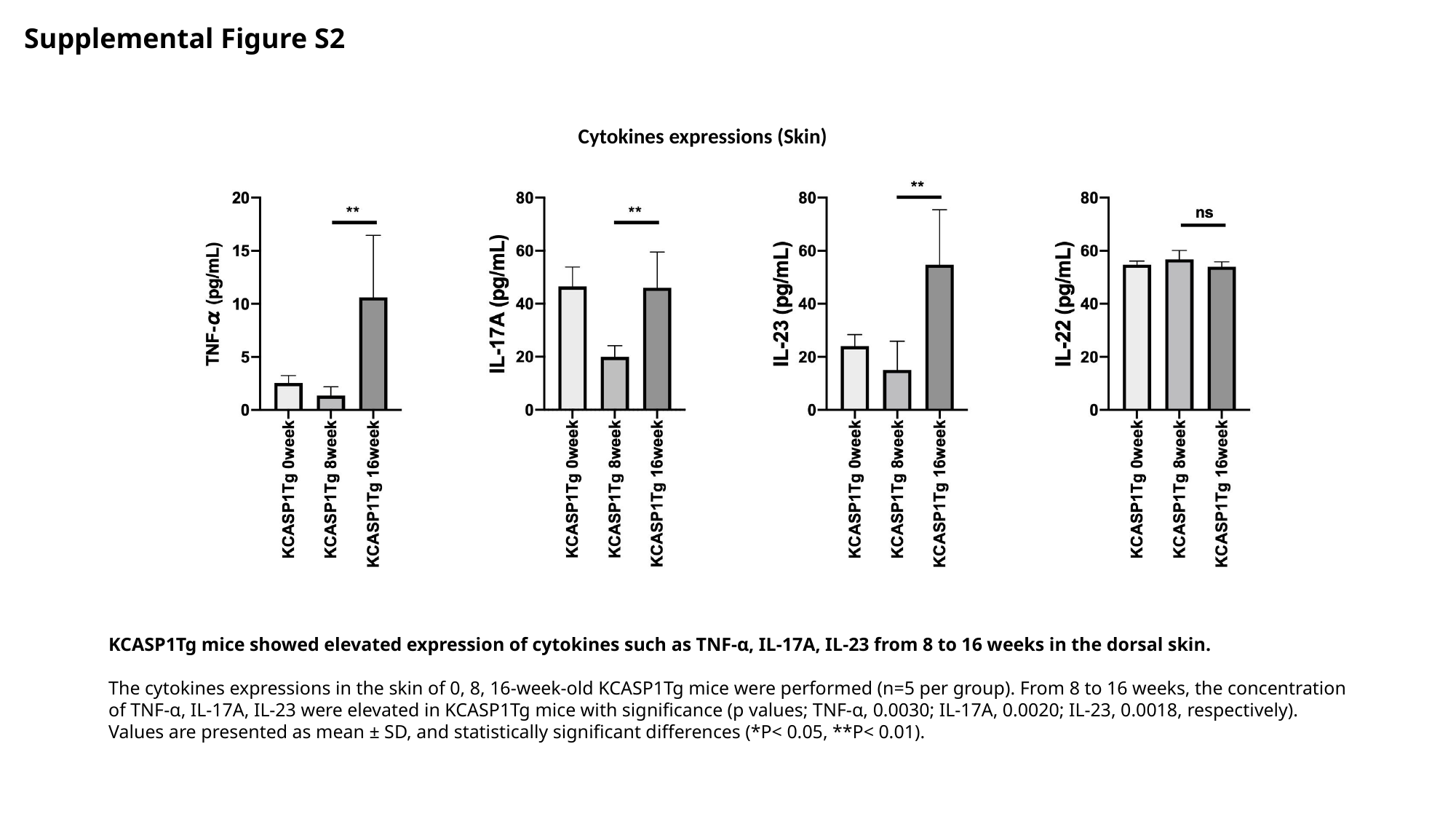

Supplemental Figure S2
Cytokines expressions (Skin)
KCASP1Tg mice showed elevated expression of cytokines such as TNF-α, IL-17A, IL-23 from 8 to 16 weeks in the dorsal skin.
The cytokines expressions in the skin of 0, 8, 16-week-old KCASP1Tg mice were performed (n=5 per group). From 8 to 16 weeks, the concentration of TNF-α, IL-17A, IL-23 were elevated in KCASP1Tg mice with significance (p values; TNF-α, 0.0030; IL-17A, 0.0020; IL-23, 0.0018, respectively).
Values are presented as mean ± SD, and statistically significant differences (*P< 0.05, **P< 0.01).

## Slide 3
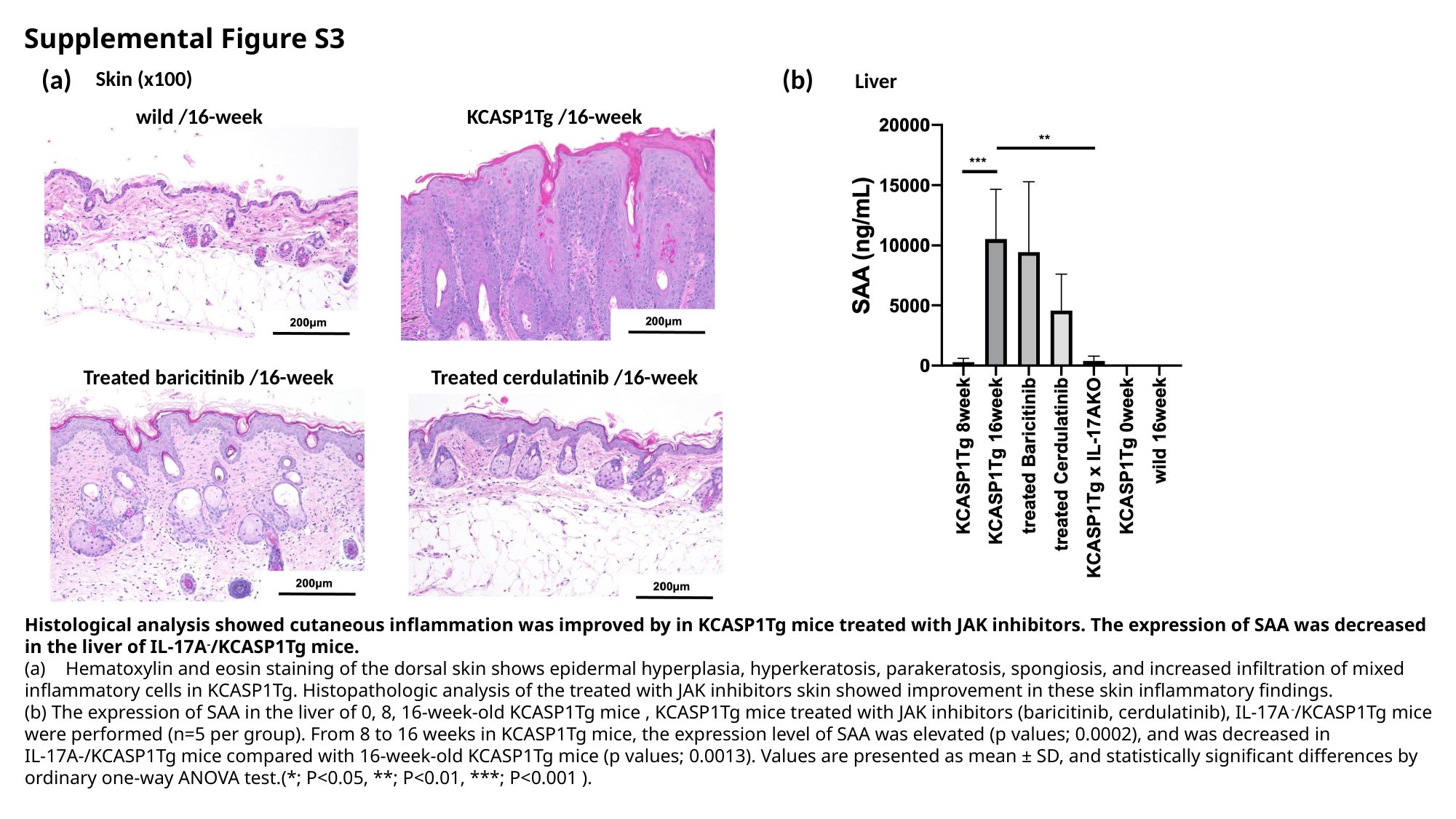

Supplemental Figure S3
(a)
(b)
Skin (x100)
Liver
KCASP1Tg /16-week
wild /16-week
Treated baricitinib /16-week
Treated cerdulatinib /16-week
Histological analysis showed cutaneous inflammation was improved by in KCASP1Tg mice treated with JAK inhibitors. The expression of SAA was decreased in the liver of IL-17A-/KCASP1Tg mice.
Hematoxylin and eosin staining of the dorsal skin shows epidermal hyperplasia, hyperkeratosis, parakeratosis, spongiosis, and increased infiltration of mixed
inflammatory cells in KCASP1Tg. Histopathologic analysis of the treated with JAK inhibitors skin showed improvement in these skin inflammatory findings.
(b) The expression of SAA in the liver of 0, 8, 16-week-old KCASP1Tg mice , KCASP1Tg mice treated with JAK inhibitors (baricitinib, cerdulatinib), IL-17A-/KCASP1Tg mice were performed (n=5 per group). From 8 to 16 weeks in KCASP1Tg mice, the expression level of SAA was elevated (p values; 0.0002), and was decreased in
IL-17A-/KCASP1Tg mice compared with 16-week-old KCASP1Tg mice (p values; 0.0013). Values are presented as mean ± SD, and statistically significant differences by ordinary one-way ANOVA test.(*; P<0.05, **; P<0.01, ***; P<0.001 ).
